# Structural Antitumoral Activity Relationships of Synthetic Chalcones

**DOI:** 10.3390/ijms10010221

**Published:** 2009-01-09

**Authors:** Cesar Echeverria, Juan Francisco Santibañez, Oscar Donoso-Tauda, Carlos A. Escobar, Rodrigo Ramirez-Tagle

**Affiliations:** 1 Instituto de Nutrición y Tecnología de los Alimentos, Universidad de Chile, El Líbano 5524, Macul, Santiago, Chile. E-Mails: cecheverrriae@gmail.com (C. E.); jfsatib@inta.cl (J. S.); 2 Universidad Andres Bello, Departamento de Ciencias Químicas, Av. República 275, Santiago, Chile. E-Mails: o.donoso@uandresbello.edu (O. D.); cescobar@unab.cl (C. E.)

**Keywords:** Antitumoral activity, DFT, structure-activity relationships

## Abstract

Relationships between the structural characteristic of synthetic chalcones and their antitumoral activity were studied. Treatment of HepG2 cells for 24 h with synthetic 2’-hydroxychalcones resulted in apoptosis induction and dose-dependent inhibition of cell proliferation. The calculated reactivity indexes and the adiabatic electron affinities using the DFT method including solvent effects, suggest a structure-activity relationship between the Chalcones structure and the apoptosis in HepG2 cells. The absence of methoxy substituents in the B ring of synthetic 2’-hydroxychalcones, showed the mayor structure-activity pattern along the series.

## 1. Introduction

Dietary flavonoids, commonly present in edible plants, are known to have beneficial effects, such as antioxidative effects, tumor cell growth inhibitory activity, and apoptosis induction in cancer cell lines. Therefore dietary flavonoids have attracted attention as chemopreventive agents [1]. Flavonoids are classified into several groups (i.e., flavonols, flavones, catechins, flavanones, chalcones, anthocyanidins, and isoflavonoids). Chalcones are the immediate precursors in the biosynthesis of flavonoids, and their structure differs considerably from the others members of the flavonoid family, since chalcones are open-chain analogs in contrast to the other family’s members.

Chalcones are abundantly present in nature starting from ferns to higher plants [2]. Chemically they are 1,3-diphenyl-2-propen-1-ones and are often cytotoxic *in vitro* [3] and some of their derivatives are reported to be antimutagenic [4]. A hydroxychalcone, isolated from *Pityrogramma calomelanos* was found to be a cytotoxic and tumor reducing agent [5]. Among flavonoids, chalcones have been identified as interesting compounds that are associated with several biological activities [6]. The most common chalcones found in foods are phloretin and its glucoside phloridzin (phloretin 2′-0-β-glucopyranoside), and chalconaringenin. Studies on the bioavailability of chalcones from food sources are limited, but synthetic chalcones have been reported to have a wide range of biological properties.

In an effort to develop a potent anti-inflammatory and cancer chemopreventive agents a series of chalcones were synthesized [7]. These compounds were tested for their inhibitory effects on the activation of mast cells, neutrophils, macrophages, and microgial cells. It is conceivable that mast cells, neutrophiles, and macrophages are important players in inflammatory disorders [7]. Activation of microglial cells also plays a crucial role in inflammatory diseases of the Central Nervous System. Thus, inhibition of the activation of these inflammatory cells appears to be an important therapeutic target for the design of new drugs for the inflammatory diseases treatment.

Particularly interesting are the properties of chalcones in the induction of apoptosis [8–9] and their ability to change mitochondrial membrane potential [10]. These authors noted that chalcones with fewer hydroxyl groups on rings A and B were more effective in this regards, as compared to chalcones containing more hydroxyl groups. This difference was attributed to the acidity of the phenolic hydroxyl groups. One of the most widely cited mechanisms by which chalcones exert their cytotoxic activity is that of interference with the mitotic phase of the cell cycle. Edwards *et al* [11] proposed a hypothetical basis for the anti-mitotic activity of chalcones. Indeed, they found a large number of methoxylated chalcones with antimitotic activity against HeLa cells. In the present work we evaluated the capacity of 2’-hydroxychalcones with different methoxy subtitutions on ring B to inhibit cellular proliferation and induce apoptosis and correlate it with the chemical reactive indexes in HepG2 hepatocellular carcinoma cells.

## 2. Results and Discussion

**Figure 1.**
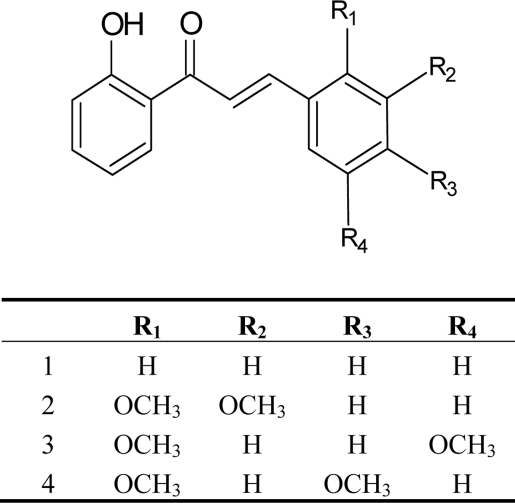
Structures of the studied chalcones.

**Figure 2.**
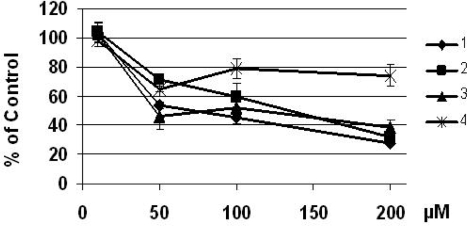
Effects of chalcones on the growth of HepG2 hepatome cells after 24 h Incubation. The results of studies are expressed as mean values ±SD from three separate experiments (-p<0.05).

In the present work synthetic 2`-hydroxychalcones containing different methoxy substitution patterns on ring B as shown in Figure 1 were tested to assess their capacity to inhibited cell growth in HepG2 hepatocellular carcinoma cells lines. HepG2 have been used successfully in a number of antitumor studies [12]. First we determined the inhibitory effects of 2`-hydroxychalcones on HepG2 cell proliferation. As shown in Figure 2, 24 h of treatment of HepG2 cells with 2`-hydroxychalcones resulted in a dose-dependent inhibition of cell proliferation. All 2`-hydroxychalcones tested inhibited cell growth at 50 μM and caused a noticeable decrease in the number of viable HepG2 cells by about 50% compared with the control, and with a higher concentration (200 μM), the viability decreased by ca. 80%, except for 2`-hydroxychalcone **4** were the cell growth augmented by ca. 50%.

With the aim to clarify whether the cell death was caused by induction of apoptosis or not, the DNA pattern and changes in nuclear morphology were examined, since DNA fragmentation and chromatin condensation are known as classical signs of apoptosis [13].

**Figure 3.**
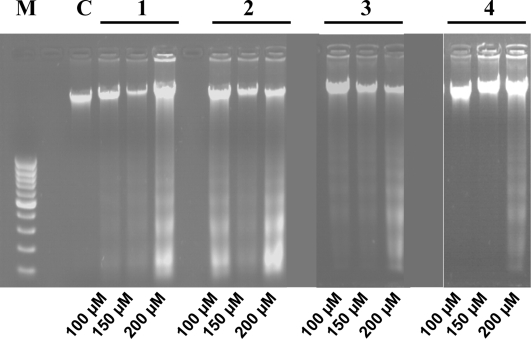
Effects of chalcones on the DNA fragmentation pattern analysis by agarose gel electrophoresis of HepG2 cells after 24 h incubation.

**Figure 4.**
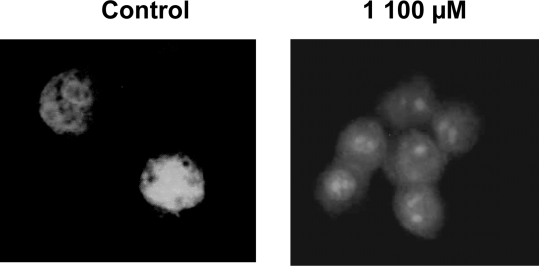
Nuclear morphology of HepG2 cells stained with ethidium bromide. The cells were incubated in the absence (control) or presence of 100 μM chalcone **1** after 24 h incubation.

To assess these, first we treated the HepG2 cells as shown in Figure 2 and we analyzed the internucleosomal DNA fragmentation. As shown in Figure 3, typical apoptosis ladder DNA patterns were observed for all four different 2`-hydroxychalcones tested. In treatments containing compounds **1** to **3** a DNA ladder were produced in all concentrations tested. In contrast, 2`-hydroxychalcone **4** only showed DNA fragmentation at 200 μM. When we analyzed the nuclear morphology under a fluorescence microscope, a chromatin condensation was evident in 2`-hydroxychalcone-treated cells compared with the control, were DNA remained dispersed in the nucleus (Figure 4). These results suggest that chalcones **1** to **3** are capable of inducing apoptosis in HepG2 cells.

**Figure 5.**
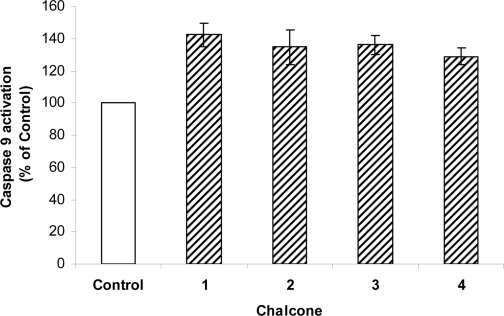
Chalcones induces activation of caspase-9. HepG2 cells treated with chalcone (200 μM) were analyzed for caspase activity after 24 h incubation. The results of studies are expressed as mean values ±SD from four separate experiments (-P<0.05).

**Figure 6.**
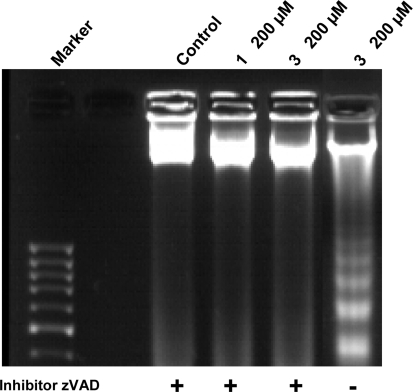
Chalcone induced apoptosis of HepG2 cells depends on the activation of caspases. Cells were treated with zVAD (50 μM) for 2 h and then incubated with chalcones **1** and **3** (200 μM) for 24 h. (cases marked by +). When the cells were incubated in absence of inhibitor (case -), typical laddering indicative of apoptosis was observed. Only representative compounds **1** and **3** are shown, but similar activity were found for all compounds tested.

The apoptosis pathway involves many factors, including caspase activation [14]. We analyzed the possibility that 2`-hydroxychalcone-induced apoptosis could be mediated by caspase activation in HepG2 cells. First we examined whether 2`-hydroxychalcone activates Caspase-9 in HepG2 cells. As shown in Figure 5, 2`-hydroxychalcone treatment (200 μM for 24 h) increased the activity level of the Caspase-9 in the HepG2 cells. In addition when HepG2 cells were tested with caspase 9 inhibitor (zVAD) in the presence of 2`-hydroxychalcones typical ladering, indicative of apoptosos induction, was not observed. (Figure 6) In contrast, in absence of inhibitor the DNA ladering was observed, suggesting that the 2`-hydroxychalcones-induced apoptosis and that this could be mediated trough the Caspase 9 pathway.

Recent research in the global electrophilicity index and the AEA (adiabatic electron affinities) show good correlations between both theoretical and experimental quantities [27, 28]. A good tendency can be observed between reactivity indexes, AEA and the antitumor activity, suggesting that the effect can be in the electron transporting chain. For this reason, we estimated the following reactivity indexes for 2`-hydroxychalcones: chemical hardness (**η**), electronic chemical potential (**μ**), electrophilicity (**ω**), and the adiabatic electron affinities (*AEA*); Results are shown in Table 1. The chemical hardness and electrophilicity decreased from compound **1** to **4**, whereas electronic chemical potential increased along the series. In relation to the adiabatic electron affinities, compound 4 showed the lower value when compared with the other compounds.

Apoptosis is one of the most potent defenses against cancer development, efforts have been made to develop a chemoprevention and therapeutic strategies that selectively trigger apoptosis in malignant cancer cells. Several flavonoids are known to inhibit cancer development *in vivo* and tumor cell growth *in vitro* [1]. Therefore they may be important in cancer chemoprevention. The present study is an attempt to investigate whether the different positions of methoxy groups can change or improve the activity of the chalcones against cancer cells proliferation. The 2`-hydroxychalcone **4** produced DNA ladders only at 200 μM (Figure 3), however, the other 2`-hydroxychalcone analogs produced DNA ladders at 100 μM, on the other hand, the cellular viability was not greater in chalcone **4** (Figure 2). These results suggest that the positions of methoxy groups in 2`-hydroxychalcone **4** resulted in a low death increase of the HepG2 cells. After treatment with 2`-hydroxychalcones, the number of HepG2 cells decreased after 24 h, suggesting the possibility of an increase in the rate of cell death. Agarose gel electrophoresis verified this suggestion (Figure 3). A DNA ladder was observed after fractionation of the nuclear DNA by agarose gel electrophoresis under UV and also chromatin condensation was observed under a fluorescence microscope (Figure 4). On the basis of these hallmarks of apoptosis, we conclude that chalcones induce apoptosis in HepG2 cells. Caspase-9 was activated by chalcones demonstrating activation in the intrinsic apoptotic pathways [25, 26] (Figure 5). The intrinsic and extrinsic apoptotic pathways converge to caspase-3, which cleaves the inhibitor of the caspase-activated deoxyribonuclease, and the caspase-activated deoxyribonuclease becomes active leading to nuclear apoptosis [25, 27]. The inhibitor of caspase-3 zVAD, eliminated the formation of ladders in all tested compounds (Figure 6). These experiments demonstrated that the caspase pathways are participating in the activation of the apoptotic events that conclude in the formation of DNA ladders, stimulated by chalcones. All our data suggests that chalcones are capable to induce apoptosis in HepG2 cells via a mitochondrial-mediated pathway, which results in the activation of caspase-9 and subsequently nuclear apoptosis.

Recent research in the global electrophilicity index and the AEA show good correlations between both theoretical and experimental quantities [28, 29]. A good correlation can be observed between reactivity indexes, AEA and the antitumor activity, suggesting that the effect can be in the electron transporting chain. This is observed clearly when comparing the activity of chalcone **4** with the rest of chalcones (Table 1). One of the electrophilic sites in the chalcone moiety can be found at the C3 carbon atom of the 2-propen-1-one moiety. The electrophilicity in this site is modulated by the presence of methoxy groups attached to the aromatic ring directly attached to the C3 carbon atom. Since the methoxy groups are electron donor substituents, they can only act stabilizing charges generated at the C3 carbon atom by mesomeric effects. This effect is expected to be similar in compounds **2** and **3**. In contrast, this stabilizing effect should be of importance in the case of compound **4**, where both methoxy groups are oriented in *ortho* and *para* positions, reducing as a consequence, the local electrophilicity at the C3 carbon atom. The absence of such substituents in compound **1**, produces the mayor electrophilicity, pattern in the series.

The calculated values of total electrophilicity are in agreement with the previously described local electrophilicity at C3 carbon atom and they are in the same trend as the biological results obtained, suggesting a relationship between the electrophilicity and the apoptotic activity of the tested 2`-hydroxychalcones in HepG2 hepatocellular carcinoma cells and the chalcone **1** would be the most active.

## 3. Experimental Section

### 3.1. Chemicals

The structures of the studied compounds **1**–**4** are given in Figure 1. The dimethoxy substituted 2’-hydroxychalcones **2**–**4** were synthesized by the Claisen-Schmidt condensation of 2`-hydroxy-acetophenone and the appropriately substituted dimethoxybenzaldehyde in basic media (KOH) as described previously by Quintana *et al*. [24] The 2’-hydroxychalcone **1** was purchased from Merck and used without further purification. These chalcones were dissolved in dimethylsulfoxide and then added to the culture medium.

### 3.2. Cell Culture

The HepG2 hepatocellular carcinoma cell line (HB 8065; American Type Culture Collection), derived from a human hepatoblastoma [30], were maintained in Dulbeccós modified Eaglés (DMEM) with 10% heat-inactivated fetal bovine serum (FBS) at 37°C with 5% CO_2._

### 3.3. Cell growth assay

Cells were seeded in a 96 wells plates at density of 10E4/well and treated by 24 h, in the last 2 h a 0.5 mg/mL of MTT at final concentration was added. Cells washed twice with PBS were treated with isopropanol-DMSO (3:2) to dissolve the formazan crystals and quantified the stain by reader a 630 nm. Cell viability is expressed as the optical density ratio of the treatment to control.

### 3.4. Internucleosomal DNA fragmentation assay

DNA was extracted from cells as described previously by Fernandes *et al* [16]. Fragmented DNA samples were separated by electrophoresis on 1.5 agarose gel and visualized with ethidium bromide.

### 3.5. Nuclear DNA condensation assay

Nuclear morphological changes in cells treated with chalcones were analyzed by ethidium bromide stain [31]. Briefly, Cells (10E5/well) were seeded in glass coverslip, and after indicates treatments cells were fixed with ice cold methanol and stained 10 min with 1 μg/mL of ethidium bromide in PBS, and after mounting in glass slice the cells nucleus condensation was observed under epi-fluorescent microscopy.

### 3.6. Activity Caspase-9

Cells were seeded in a 96 wells plate at density of 10E4/well, the activity caspase-9 was analyzed using the Caspase-Glo® 9 Assay (Promega) kit according the manufacturing instructions.

### 3.7. Section model equations

The global electrophilicity index ω, which measures the stabilization in energy when the system acquires an additional electronic charge ΔN from the environment, is been given by the following simple expression due to Parr *et al.* [16, 17]:

(1) $$\omega \;=\;\mu^{2}/\;2\eta \;$$

where in terms of the electronic chemical potential μ and the chemical hardness η, ω may be approximated in terms of the one electron energy of the frontier molecular orbital HOMO and LUMO Є_Í_ and Є_L,_ as μ ≈ (Є_Í_ + Є_L_)/2 and η ≈ Є_Í_ – Є_L_ respectively [16,17]. The electrophilicity index encompasses both the tendency of the electrophile to acquire an additional electronic charge driven by μ^2^ (the squared of the chemical potential) and the resistance of the system to the exchanging electronic charge with the environment described by η.

The electron affinity is defined as the total energy of the neutral Chalcones minus that of the anion radical (*E*° - *E*-). The calculation of the adiabatic electron affinity (AEA) is based on the optimized geometry of the neutral species and the optimized geometry of the anion radical species.

### 3.8. Computational Details

Our calculations were carried out by using the Amsterdam Density Functional (ADF) code [18]. All the molecular structures were fully optimized via the analytical energy gradient method implemented by Verluis and Ziegler employing the local density approximation (LDA) within the Vosko-Wilk-Nusair parameterization for local exchange correlations [20, 21]. We also used the GGA (Generalized Gradient Approximation) BLYP and hybrid B3LYP functional [19]. Solvation effects were modeled by a conductor-like screening model for real solvents (COSMO) [22, 23] using water as solvent. The cluster geometry optimization and the energies were calculated using a standard Slater-type-orbital (STO) basis sets with triple-zeta quality with double polarization functions (TZ2P) for all the atoms. The global electrophilicity (ω) was evaluated using eq 1. The electronic chemical potential μ and the chemical hardness η were evaluated using the frontier molecular orbital model described in the Model Equations Section.

## 4. Conclusions

Treatment of HepG2 cells for 24 h with synthetic 2’-hydroxy-Chalcones resulted in a dose-dependent inhibition of cell proliferation and apoptosis induction. The calculated reactivity indexes and the adiabatic electron affinities using the DFT method including solvent effects, suggest a structure-activity relationship between the 2’-hydroxy-chalcones structure and the apoptosis in HepG2 cells.

## Figures and Tables

**Table 1.** Reactivity Indexes; chemical hardness (**η**), electronic chemical potential (**μ**), electrophilicity (**ω**) and the adiabatic electron affinities (***AEA***). Values are given in (Kcal/mol).

Compound Nº	η	μ	ω	*AEA*
1	84.63	−42.20	142.28	72.52
2	83.71	−41.74	137.21	73.40
3	78.41	−39.20	113.69	73.96
4	75.87	−37.81	102.39	69.64
